# Understanding the acceptability of e-mental health - attitudes and expectations towards computerised self-help treatments for mental health problems

**DOI:** 10.1186/1471-244X-14-109

**Published:** 2014-04-11

**Authors:** Peter Musiat, Philip Goldstone, Nicholas Tarrier

**Affiliations:** 1 King’s College London, Institute of Psychiatry, De Crespigny Park, PO-Box 077, London SE5 8AF, UK

**Keywords:** E-mental health, M-health, Computerised CBT, Internet, Smartphones, Mental health

## Abstract

**Background:**

E-mental health and m-mental health include the use of technology in the prevention, treatment and aftercare of mental health problems. With the economical pressure on mental health services increasing, e-mental health and m-mental health could bridge treatment gaps, reduce waiting times for patients and deliver interventions at lower costs. However, despite the existence of numerous effective interventions, the transition of computerised interventions into care is slow. The aim of the present study was to investigate the acceptability of e-mental health and m-mental health in the general population.

**Methods:**

An advisory group of service users identified dimensions that potentially influence an individual’s decision to engage with a particular treatment for mental health problems. A large sample (*N* = 490) recruited through email, flyers and social media was asked to rate the acceptability of different treatment options for mental health problems on these domains. Results were analysed using repeated measures MANOVA.

**Results:**

Participants rated the perceived helpfulness of an intervention, the ability to motivate users, intervention credibility, and immediate access without waiting time as most important dimensions with regard to engaging with a treatment for mental health problems. Participants expected face-to-face therapy to meet their needs on most of these dimensions. Computerised treatments and smartphone applications for mental health were reported to not meet participants’ expectations on most domains. However, these interventions scored higher than face-to-face treatments on domains associated with the convenience of access. Overall, participants reported a very low likelihood of using computerised treatments for mental health in the future.

**Conclusions:**

Individuals in this study expressed negative views about computerised self-help intervention and low likelihood of use in the future. To improve the implementation and uptake, policy makers need to improve the public perception of such interventions.

## Background

In recent years, computerised interventions for mental health problems have been increasingly investigated as alternatives or adjunct to usual care. Electronic and mobile mental health interventions (e-mental health/m-mental health) are available for depression and anxiety [1,2], eating disorders [3], substance use disorders [4], and medically unexplained conditions, such as tinnitus [5] or irritable bowel syndrome [6]. Despite the increasing evidence base for the efficacy of such interventions, the transition and implementation of these into clinical practice is remarkably slow [7]. In the UK, only two computerised interventions for mental heath problems are recommended in the clinical treatment guidelines (i.e. Beating the Blues for depression, Fear Fighter for panic and phobia; [8,9]). Hence, it appears that amongst healthcare providers and potential service users there are barriers to the implementation of effective computerised interventions that have not yet been fully identified or addressed. We propose that the acceptability of computerised interventions may be one key barrier.

It is often assumed that e-mental health interventions are associated with a number of benefits over traditional face-to-face care. These are the increased convenience for the patient with regards to time and location of treatment, the anonymity of such interventions, the reduced costs for healthcare providers and the ability to bridge gaps in the provision of care (e.g. [10-12]). Although there is evidence suggesting that individuals with mental disorders perceive these aspects of e-mental health as potentially advantageous [13] it is unclear how this affects an individual’s decision to engage with such interventions. In addition, we have recently found that the current evidence base for these added benefits or ‘collateral outcomes’ is sparse [14].

Within randomised controlled trials, high acceptability of computerised mental health interventions has been reported, but these results primarily stem from individuals who completed a course of treatment [15]. Hence, this is likely to be an overestimate given the high dropout rates often observed in computerised intervention trials (for a review, see [16]). In their review of barriers for the uptake of computerised cognitive behaviour therapy, Waller and Gilbody [17] noted that with regard to research dropout, individuals receiving computerised CBT were twice as likely to dropout as those receiving face-to-face CBT. The authors conclude that “acceptability is high among those who make it as far as participating in studies, but initial uptake rates suggest that this may not be the whole picture” ([17], p. 709).

More generalizable information stems from studies investigating individuals’ views on computerised treatments outside the context of intervention trials. In a study with patients with obsessive-compulsive disorder, Wootton et al. [13] found that only 22% of patients believed that online therapy would improve their symptoms substantially, whereas the majority only assumed small (59%) or no improvements (16%). With respect to the disadvantages of internet therapy, 10% patients reported preferring face-to-face treatment and 9% thought their “problems are too complex or severe to be treated online”. Data on face-to-face CBT were not assessed in this study and the results stem from a sample visiting a national internet treatment website (http://www.virtualclinic.org.au). This study highlights that even in a sample of patients willing to visit an online therapy website, there are concerns to be addressed. Patient responses in a qualitative study of the acceptability of computerised CBT (cCBT) in depression suggested that the computerised intervention could aggravate existing social isolation and that it needs more human interaction [18]. Gun et al. [19] conducted a survey with non-health professionals and health professionals and both professionals and lay people reported being slightly positive towards computerised therapy. However, those with more severe symptoms reported negative attitudes towards cCBT. Participants were recruited from a website, which suggests again that the sample may have been biased towards individuals with good access to technology and an interest in computerised interventions.

More recently, Carper et al. [20] investigated patients’ and clinicians’ perceptions of computerised treatments in the context of theories of dissemination and implementation. Although patients did not perceive computerised therapy as more complex to use, they also did not perceive any relative advantage over other forms of care. In addition, they reported low ‘observability’ (i.e. familiarity) scores, suggesting that they perceive the intervention as not frequently used or did not know anyone who has used it in the past. As a result, patients reported low intentions for future use of computerised therapy. The authors concluded that increasing ‘observability’ is key to increase adoption rates [20]. However, the sample in this study was small (*n* = 55) and consisted only of individuals seeking treatment for anxiety and depression.

Results from previous studies highlight that there is a lack of large-scale investigations into the acceptability of computerised therapy for mental health. Hence, the aim of this study was to address this limitation by conducting a survey in a broad population comparing the acceptability of computerised self-help with face-to-face treatment and bibliotherapy. Bibliotherapy was included, as it is a popular type of self-help and to compare computerised self-help against self-help that is not technology-mediated. In addition, this study aimed to identify aspects of mental health treatments that may influence an individual’s decision to engage with a treatment.

## Methods

### Sample

Participants were recruited via email circulars, social networks, through flyers in public places, and on a national mental health internet forum. Individuals were considered eligible if they were aged 18 or older and there were no exclusion criteria. The study was advertised as a survey on the attitudes and expectations toward different treatment options in mental health. No reference to technology or self-help was made, as this may have potentially deterred some individuals from participating. In addition, participants had the option to enter into a raffle for an online shopping voucher (£100). Ethical approval for the study was given by the King’s College London Psychiatry, Midwifery and Nursing Research Ethics Sub-committee (REF PNM/12/13-137). The recruitment material for this study included a link to the study website, where individuals could get more information about the aims of the study. After providing informed consent, participants were able to complete the online questionnaires.

### Measures

Questionnaires for this study were developed in collaboration with the Service User Advisory Group of the NIHR Biomedical Research Centre at South London and Maudsley NHS Foundation Trust and King’s College London. This group includes individuals with experience of mental health problems and mental health services, who offer advice on the design and management of research projects. In a focus group, individuals were asked to identify aspects or qualities of mental health treatments, which may influence their decision to engage in a particular treatment option for mental health problems. Twelve domains were identified, which are shown in Table 1 and formed the basis for the questionnaires used in this study. Participants in the survey were asked to provide general demographic information and information about previous or current, diagnosed or undiagnosed mental health problems, as well as help-seeking behaviour. To investigate expectations towards mental health treatment, individuals were asked to rate the importance of each of the 12 domains on a 7-step rating scale ranging from 0 “not important at all” to 6 “very important”. For this question, participants were instructed to think of mental health treatments in general and what they would consider important if they were to seek help right now. This was followed by three questions asking participant to indicate to what extent they think different treatment options for mental health problems would meet their expectations with regard to the 12 domains identified by service users. The treatments compared were face-to-face treatment (including, but not limited to CBT), bibliotherapy, web-based interventions and mental health applications for smartphones. Each type of treatment was briefly explained with a few sentences, as it was possible that not all participants were familiar with the different options. Expectation ratings in these questions were obtained on a 7-step rating scale ranging from 0 “Would not meet my expectations at all” to 6 “Would fully meet my expectations”. In addition, participants were asked to rate how likely they would use any of the investigated treatment options on a 5-step rating scale from 0 “Very unlikely” to 3 “Very likely”.

**Table 1:** Evaluation dimensions for mental health treatments

	
Helps with the problem	Motivates to get better
Is credible	Is accessible without waiting time
Appeals	Can be accessed at a convenient time
Is free of charge	Can be accessed at a convenient location
Can be accessed anonymously	Includes personal support
Provides feedback	Suits own learning style

Finally, to assess computer literacy and familiarity with technology, participants were asked to indicate how frequently they use different forms of technology, such as computers in general, internet, smartphones or tablets, on a 5-step rating scale. Responses were added up to form a general computer literacy score.

### Statistical analysis

A repeated measures MANCOVA was conducted to investigate whether individuals have different expectation for different treatment options with regard to the 12 domains identified by service users and their likelihood of use for each intervention. The composite computer literacy score was included as a covariate and gender, previous mental disorders, current mental disorders and help-seeking were included as between-subject effects. Post-hoc comparisons were used to investigate differences between particular treatment options.

## Results

A total of 617 individuals accessed the online questionnaire website and provided consent. However, only 490 (79%) of participants completed enough questions to be included in the analysis (i.e. less than 10% of items missing). Table 2 shows the demographic characteristics of the sample. A wide age range of participants was recruited, however, the average age of participants was 27 years. Participants were primarily female, students and had at least an undergraduate university degree. In addition, ethnic diversity in the sample was low with the majority of the sample identifying as White British. Two hundred and forty (49%) reported having had mental health problems in the past and 107 (22%) participants reported currently suffering from a mental health problem. Of those with a history of mental health problems, 85% had sought help and 60% had been formally diagnosed.

**Table 2:** **Demographic characteristics of study participants (*N*** **= 490)**

	
Age:	
Range	18-78
*M* (*SD*)	26.7 (8.9)
Sex: *n* (%)	
Female	383 (78.2)
Male	105 (21.4)
Missing	2 (0.4)
Ethnicity: *n* (%)	
White British	385 (78.6)
Asian/Asian British	60 (12.2)
Black	
Mixed/Multiple	17 (3.5)
Other	22 (4.5)
Employment status: *n* (%)	
Full time	143 (29.2)
Part time	35 (7.1)
Unemployed	6 (1.2)
Student	290 (59.2)
Sick leave	1 (0.2)
House wife/husband	1 (0.2)
Other	14 (2.9)
Education: *n* (%)	
No qualifications	1 (0.3)
O Level/GCSE	4 (1.2)
A Level/NVQ	
Diploma/BTEC	16 (3.3)
University degree	173 (35.3)
Postgraduate degree	149 (30.4)
Other	16 (3.3)
Marital status: *n* (%)	
Married	68 (13.9)
Living together	83 (16.9)
Single	335 (68.4)
Divorced	4 (0.8)

### Expectations towards mental health treatments

Participants were asked to rate the importance of each aspect identified by the group of service users and the results are shown in Table 3. On the seven-step rating scale ranging from 0 “not important at all” to 6 “very important”, all aspects were rated highly important. However, participants rated the helpfulness and credibility of an intervention as most important, alongside with the ability of an intervention to motivate and the ability to access it without waiting time. The anonymity of an intervention and whether it suits one’s individual learning style were rated least important.

**Table 3:** Importance ratings for 12 dimensions in order of importance (highest on top)

**Dimension**	**Importance *M *** **(*SD*)**
Helps with the problem	5.77 (0.52)
Motivates to get better	5.35 (0.93)
Is credible	5.35 (0.86)
Is accessible without waiting time	5.33 (0.95)
Can be accessed at a convenient time	4.99 (1.10)
Provides feedback	4.93 (1.20)
Includes personal support	4.87 (1.26)
Can be accessed at a convenient location	4.85 (1.13)
Is free of charge	4.75 (1.33)
Appeals	4.70 (1.28)
Suits own learning style	4.48 (1.40)
Can be accessed anonymously	4.16 (1.71)

### Acceptability of different interventions

**Figure 1:**
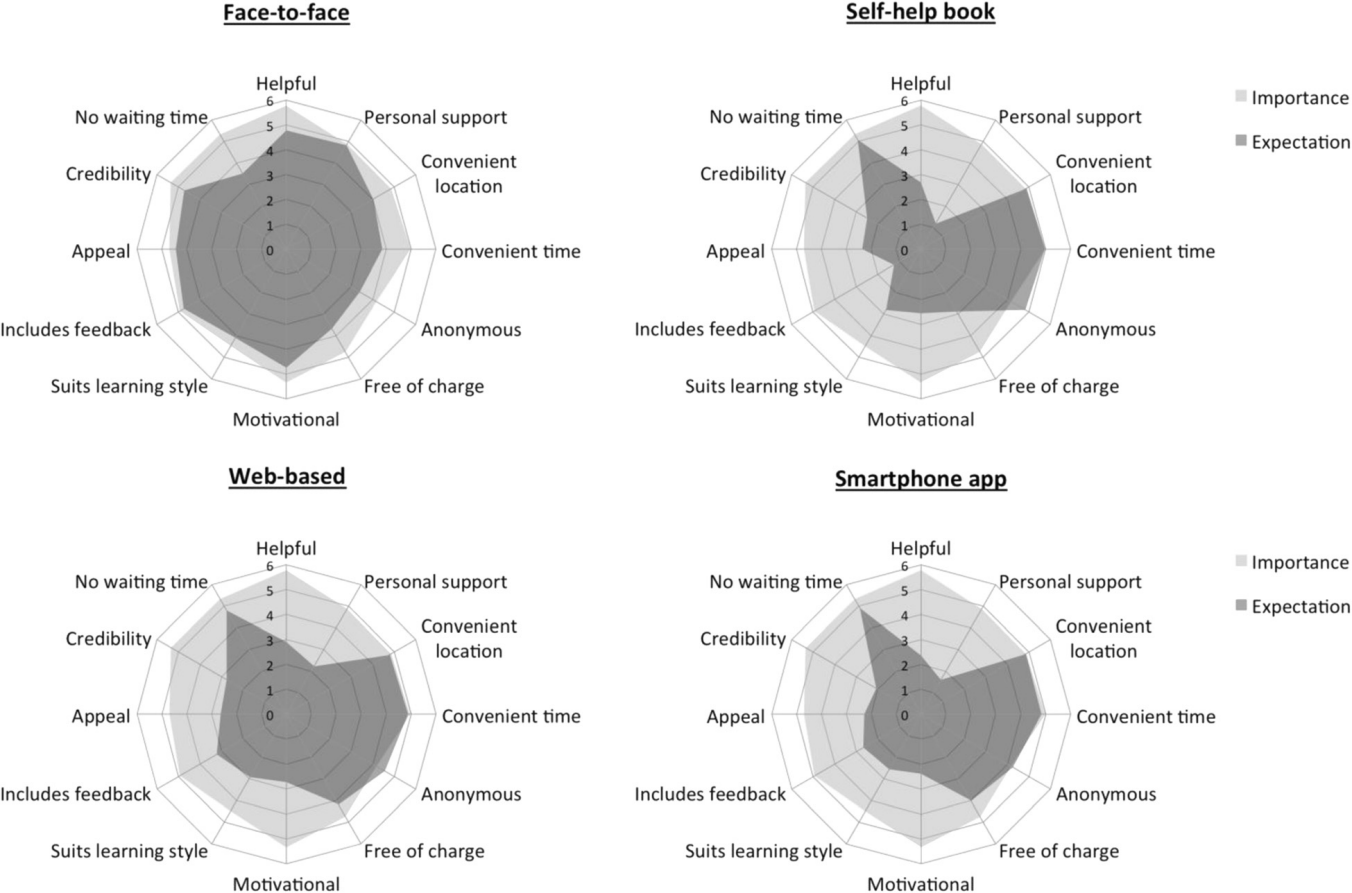
Importance and expectations towards different mental health treatment options.

Participants were asked to rate to what extent they think a particular treatment for mental health problems meets their expectations on each of the 12 domains. Figure 1 shows the match/mismatch between the importance of each domain and participants’ ratings of how much they thought each treatment would meet their expectations. Multivariate analysis with a repeated measures MANCOVA revealed a significant within effect for intervention type, indicating that participants reported different acceptability and likelihood of use for each intervention (*V* = 0.33, *F* (39,355) = 4.46, *p* < .001, *η*2 = 0.33). There was no significant effect for gender (*V* = 0.05, *F* (13,381) = 1.42, *p* = .148), previous mental health problems (*V* = 0.03, *F* (13,381) = 1.00, *p* = .452), current mental health problems (*V* = 0.03, *F* (13,381) = 0.89, *p* = .568), or previous help-seeking (*V* = 0.02, *F* (13,381) = 0.48, *p* = .934).

Across all domains, participants expected face-to-face therapy to meet their expectations to a medium or high extent. Face-to-face therapy was perceived to be helpful, appealing, and include feedback and personal support. Lower expectations were reported for the convenience of access, anonymity, waiting time for treatment and potential costs associated with face-to-face treatment. Self-help books were rated highly with regard to meeting participants’ expectations towards convenience of access, anonymity and waiting time. On other dimensions, participants indicated that their expectations would not be met by a self-help book. Lowest ratings were reported on the domains of personal support and feedback. Similarly to self-help books, participants indicated a high degree of their expectation being met for convenience of access, waiting time for treatment and anonymity for web-based mental heath interventions. In addition, participants anticipated web-based interventions likely to be free of charge. Web-based interventions were only expected to moderately meet participants’ expectations with regard to helpfulness, credibility, appeal, feedback and learning style. Smartphone applications for mental health problems were expected to be conveniently accessible, anonymous, free of charge and accessible without waiting time. However, ratings on all other dimensions were low.

Post-hoc comparisons revealed significant differences between interventions on all dimensions of acceptability. With regard to helpfulness, personal support, motivation, learning style, feedback, appeal and credibility, participants reported that face-to-face therapy is more likely to meet their expectations on these domains than any computerised therapy or self-help books. On the domains of treatment access at a convenient time and from a convenient location, participants indicated that face-to-face treatment is least likely to meet their expectation. On these two dimensions, all self-help interventions received significantly higher ratings and there was no difference between self-help books, web-based interventions or smartphone apps. Participants rated self-help books and web-based interventions as most likely to meet their expectations with regard to anonymity, followed by smartphone apps and face-to-face interventions. Ratings on the dimension “is free of charge” suggested that web-based interventions and smartphone app were most likely to meet individuals’ expectations. Lowest ratings on this dimension were reported for face-to-face treatment for mental health problems. Regarding the waiting time for treatment, participants indicated that self-help interventions would more likely meet their expectations than face-to-face treatment. No differences between self-help books, web-based interventions and smartphone apps were observed on this domain.

### Likelihood of use

**Figure 2:**
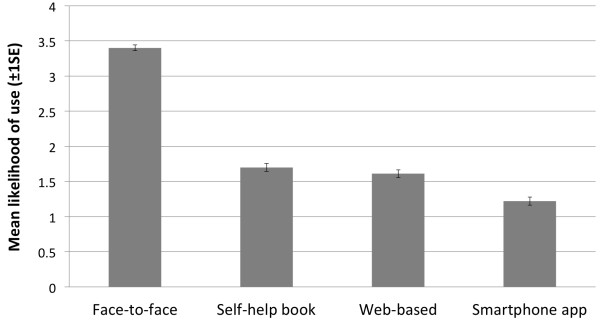
Likelihood of use for face-to-face therapy and self-help interventions.

Participants were asked to indicate how likely they would use each intervention type on a five-step rating scale ranging from “very unlikely” to “likely”. Univariate tests revealed significant differences in likelihood of use between the conditions (*F* (2.73) = 17.39, *p* < .001). Figure 2 shows the likelihood of use ratings for each intervention. Post-hoc comparisons revealed that participants were most likely to access face-to-face therapy for mental health. There was no significant difference between the likelihood of use between self-help books and web-based interventions. Participants rated the use of smartphone apps for mental health problems as least likely and compared to all other interventions and this difference was significant. No significant differences on the likelihood of use between interventions were observed with regard to gender, previous mental health problems or current mental health problems. Individuals that reported suffering from a mental health problem in the past and had sought help for this problem, reported a lower likelihood of use for smartphone interventions (*t* (447.34) = 2.144, *p* < .05, r = .10).

### Computer literacy

A large majority of participants indicated using computers several times a day (94.5%) or at least once a day (4.3%). Similarly, 97.5% of participants reported using the internet several times a day. Eighty six per cent of participants indicated using a mobile phone several times a day and 86% indicated using a smartphone several times a day, suggesting that a majority of the sample owns a smartphone device.

Computer literacy was included as a covariate in the analysis of intervention acceptability and showed a significant main effect on expectance ratings and likelihood of use (*V* = 0.08, *F* (13,381) = 2.67, *p* < .01, *η*2 = 0.08). However, a significant interaction between computer literacy and intervention type was not observed (*V* = 0.13, *F* (39,355) = 1.32, *p* = .100). Computer literacy was weakly, but significantly correlated with age (*r* = −.130, *p* < .01), indicating that higher age was associated with lower computer literacy. A median split was performed to investigate whether computer literacy affects the likelihood of use for a particular intervention. Individuals with higher computer literacy reported a significantly higher likelihood (*M* = 1.37, *SD* = 1.27 compared to *M* = 1.08, *SD* = 1.24) of using a smartphone app for mental health problems (*t* (485) = −2.581, *p* < .05, *r* = .12), there were no differences between groups with regard to the likelihood of use for other interventions.

## Discussion

The present study investigated attitudes and expectation towards different treatment options in mental health, in particular face-to-face treatment, bibliotherapy, web-based treatments and mental health treatment apps. Although recruitment was broad, the sample consisted primarily of female university students. The acceptability of different treatment options was investigated with regard to 12 domains identified in collaboration with service users.

Unsurprisingly, participants identified the helpfulness of an intervention for mental health problems as the most important criterion with regard to making treatment choices. Similarly to the study by Wootton et al. [13], it appears that participants consider self-help interventions less likely to be helpful. One possible reason for this perception is that participants closely associate the perceived helpfulness of face-to-face therapy with the amount of personal support received, as well as the fact that it is provided by a health professional. In this study, personal support was rated second-most important with regard to seeking help for mental disorders. Although it is relatively well established that mental health service users highly value the personal component of face-to-face therapy [21], it is possible that the high importance ratings found in this study are somewhat an artefact of directly comparing self-help treatments against face-to-face therapy.

The fact that individuals considered convenience with regard to time and location of treatment as an important criterion is in accordance with previous research [21]. Surprisingly, this is not reflected in the likelihood of use rating, in which participants indicated low readiness to engage in web-based therapy or mental health smartphone applications. This is somewhat contradictory to individuals’ responses with regard to the importance of each dimension. Participants identified the low treatments costs, convenience of access and short waiting times as highly important domains when it comes to seeking help for mental health problems and also perceived self-help as superior on these domains than face-to-face therapy.

Anonymity is often quoted as one of the advantages of computerised forms of self-help [22] and it appears that users of such interventions share that opinion [23]. The results from this study paint a different picture. Of the 12 dimensions identified by service users as important for decision-making, anonymity was rated least important. In addition, although self-help treatments were perceived as significantly more anonymous, face-to-face therapy did not score particularly low on this domain. Similar to recent evidence by Carper et al. [20], it is possible that anonymity is simply not perceived as a particular advantage of computerised self-help.

Unsurprisingly, computer literacy was generally high in this sample, given the high proportion of university students and the low medium age. A clear impact of computer literacy on the acceptability or likelihood of use of different interventions as suggested by the literature ([24] could not be observed in this study. However, it is possible that this was due to the relatively large homogeneity of the sample with regard to age and ethnicity, as these are factors assumed to be associated with computer literacy. In the present study, owning a smartphone per se did not seem to increase the likelihood for using mental health interventions on such devices, given that the majority of participants appeared to own smartphones. With 86% of the sample using smartphones several times a day, the rate is considerably higher than the national average, which suggests that only 48% use smartphones [25]. However, higher computer literacy in general (the frequent use of different technologies and devices) slightly increased the reported likelihood for the use of smartphones for mental health interventions.

The way participants were asked about the acceptability of self-help interventions may have influenced their acceptability ratings. Given the inclusion of face-to-face interventions, participants may have perceived this intervention as the benchmark for a treatment and subsequently rated self-help interventions as less acceptable. In addition, participants may have felt that self-help interventions were suggested as an inferior replacement to face-to-face therapy. However, this is not necessarily the case. Self-help books and web-based interventions for common mental disorders exist that achieve similar effects in randomised controlled trials compared with face-to-face therapy (e.g. [26,27]) and therefore could be an alternative treatment for those not willing or able to access face-to-face treatments. Moreover, self-help can be a first step in treatment for mild to moderate cases as seen within the stepped care approach recommended in NICE, or to bridge the time between contact with services and the beginning of treatment.

Individuals’ views on different treatment options are likely to be affected by the amount and quality of information provided on these options. In this study, only brief descriptions were provided for each treatment, to obtain an unbiased view on the acceptability of face-to-face and self-help interventions. In addition, as e- and m-mental health interventions are often designed to be accessible without contact to a health care professional, individuals will likely have only very limited information available and have to base their decision on whether to engage with the treatment on these information.

Given the polarised and highly correlated responses, it is difficult to disentangle which dimensions in particular influence an individual’s decision to engage with a mental health treatment, such as face-to-face therapy or self-help. In this study, individuals were asked to rate the importance of different aspects in general and not with regard to a particular treatment. This somewhat hypothetical scenario may have contributed to the inconsistencies in individuals’ responses. It is possible that the importance of each dimension is highly specific to the disorder and also dependent on environmental circumstances within the individual, such as geographical distance to services, disabilities or conditions preventing access, waiting times or stigma. Faced with the evidence as well as the choice of trying one form of self-help or waiting for treatment to become available, many service users might engage with computerised interventions, despite their concerns. The dimensions identified in this study could be used in future studies to investigate which of the dimensions predict behaviour with regard to engaging in health interventions.

### Strengths and limitations

This study is one of the few studies that investigated the acceptability of computerised (and other) self-help for mental disorders in a general population. The sample was large, included individuals from all ages and was recruited using different strategies. Acceptability was investigated on domains that were identified by service users as important aspects of mental health treatment. This sets the current study apart from previous research and ensured that the obtained data has high ecological validity. However, ethnic minorities were underrepresented in this study, and this group often has less access to computer technology [24]. In addition, data collection was conducted online. As a consequence, participants had to have access to a computer as well as a certain degree of computer literacy to participate in the study. This may have biased the results and it is possible that acceptability ratings for computerised forms of self-help are overestimated.

## Conclusion

The results from this study may appear anticlimactic, as current and potential service users do not seem to share the enthusiasm with regard to using computerised interventions. However, this may rather be reflecting a perception of computerised interventions as an inferior and less helpful mode of treatment than face-to-face treatment delivered by a health professional. Particularly as this study has highlighted that it is the perceived helpfulness of an intervention that appears to be an important factor influencing the likelihood of use. The results also suggest that individuals are well aware of the potential advantages of computerised interventions, such as short waiting times and convenient access, but may not place importance on these factors. Thus, this study highlights the important need to raise awareness amongst clinicians and service users (most likely via these clinicians) about the growing evidence base for computerised (including mobile) mental health interventions. For e-mental health to have the large public health impact that it is often praised for, there is a need for improving the translation of e-health research into clinical practice. Steps towards achieving this may include not only making clinicians and subsequently service users aware of the evidence for these interventions, but also increasing the inclusion of the evidence-base and efficacy of computerised mental health interventions into clinical treatment guidelines. As the attitudes towards different treatment options for mental health are likely subject to the amount and quality of information available to the user, an effort should be made in improving the image of e- and m-mental health, as well as providing users with sufficient information to make an informed choice.

## Competing interests

The authors declare that they have no competing interests.

## Authors’ contributions

PM conceived and designed the study, contributed and supervised data collection, analysed and interpreted the data, and drafted the manuscript. PG collected data, contributed to the analysis and interpretation of the data, as well as the drafting of the manuscript. NT contributed to the design of the study and performed the critical revision of the intellectual content. All authors read and approved the final manuscript.

## Pre-publication history

The pre-publication history for this paper can be accessed here:

http://www.biomedcentral.com/1471-244X/14/109/prepub
